# Promoting culturally informed and sensitive practice in day-care centers—a contribution to the professionalization of day-care teachers

**DOI:** 10.3389/fpsyg.2023.1192631

**Published:** 2023-08-03

**Authors:** Ursula Gisela Buchner, Constanze Maria Luise Eberl, Markus Hess

**Affiliations:** ^1^DHGS Deutsche Hochschule für Gesundheit und Sport, Ismaning, Germany; ^2^DHGS Deutsche Hochschule für Gesundheit und Sport, Berlin, Germany

**Keywords:** professionalization, day-care teachers, day-care center, intercultural sensitivity, intercultural competence, early childhood education

## Abstract

**Background:**

Based on the growing number of families and young children with a refugee background in Germany, day-care teachers face enormous challenges regarding culturally informed practice. The project “Gemeinsam stark durch den Start” (Stronger together by starting together) addresses these challenges on various levels. At the level of day-care teachers, training in culturally informed and sensitive education is combined with reflection sessions about their own attitudes and prevailing intercultural norms, thus contributing to the professionalization of day-care teachers.

**Aim:**

This paper focuses on mechanisms of action that contributed to the effectiveness of the training from two perspectives: the day-care teachers’ perspective and the trainers’ perspective.

**Methods:**

Staff members of 11 German day-care centers underwent graded online training sessions (team and in-depths trainings) addressing intercultural topics. All participants were presented with a questionnaire for their training evaluation before and after the training sessions. Also, participants of the in-depths trainings participated in semi-structured interviews on the training. Furthermore, qualitative interviews were conducted with all trainers (*N* = 4) of the workshops.

**Results:**

Day-care teachers evaluated the online training positively, especially the improvement of professionalization and the implementation of training elements. Results reveal that (work-related) reflexive sessions as well as sessions dealing with the implementation of exercise tools into daily practice were rated as fundamental parts in the training. Teachers from high-risk day-care centers estimated the trainings’ effectiveness lower than those working in low-risk day-care centers. Qualitative data shows that the day-care teachers are in need of (theoretical) knowledge about all training elements and hands-on advice for dealing with specific situations. Especially day-care teachers within a high-risk environment, who already report having an elevated level of intercultural knowledge and skills, may need a higher dose training while low-risk day-care teachers may profit more from a low threshold training.

**Conclusion:**

The introduced training sessions focusing on intercultural sensitivity and competence present an important contribution to the professionalization of day-care teachers in working with children from different cultural backgrounds. Trainings should focus on reflexive elements as well as exercises in perspective taking and provide hands on materials for daily work.

## 1. Introduction

In spite of Germanys consolidated status as an immigration country, social institutions are still not sufficiently prepared for people with a migration background, especially those with a displacement or refugee experience. In the past years, a number of crises such as the war in Syria or the ongoing war in Ukraine have led to a rise in numbers of families with young children with a refugee background. A substantial number of refugees experience traumatizing events before or during their flight. These can be particularly severe for children and may have a negative impact on their development including school achievement (Hasselhorn et al., 2014), as well as causing physical and psychological impairments (Fingerle and Wink, 2020). Furthermore, families face challenges such as learning to navigate new surroundings and authorities as well as learning a new language and culture. Regarding aversive health effects, research for a long time focused on pre-migration traumata that might result in health problems after resettlement in new host societies (Chantler, 2012). Only recently a shift in attention took place where scientists more strongly investigated post-migration risk factors for refugees. Studies from the United Kingdom for example have shown that refugees face economic and social stressors in their host countries, such as unemployment, poverty, uncertainty about residency, social isolation, inadequate housing, discrimination, and language difficulties (see James et al., 2019). Therefore, it is important to include a discussion of post-migration risk factors as structural factors that might mitigate efforts to implement intercultural sensitivity in early childhood education (ECE).

Regarding integration into new host societies, one has to consider differences in norms and values that prevail in families shaped by original cultural backgrounds which might at least partially promote or hinder the acculturation process (van der Zee and van Oudenhoven, 2022). Although beyond the scope of this paper, it should be mentioned that concepts of intercultural education and intercultural sensitivity should consider the challenges and contradictions of multicultural contexts related to the ongoing debate about cultural relativism versus universalism (Schilmöller, 2009).

Regardless of the reason for migration, it leads to changes in social and family networks and it is linked to various social topics such as the integration of children with a refugee background into the German educational system (Geisen et al., 2014). Especially for young children it is important to establish a safe environment to promote an appropriate cognitive and social–emotional development (Britto et al., 2017). With regard to the aforementioned risk factors, it is important to provide a culturally informed professional environment in early institutional care to support children in families who often struggle with a number of adjustments to the new host society (Belhadj Kouider et al., 2014).

Hence, teachers in early institutional day-care experience issues of establishing innovative and culturally informed practices to create the premises for needs-oriented integration. This requires a high level of professionalism (Haslip and Gullo, 2018). Professional practice is characterized by the amalgamation of scientific and practical knowledge and allows professionals to develop new and appropriate approaches and actions (Dewe and Otto, 2015). Furthermore, difficult situations require negotiation and actions skills and the ability to self-reflect when processing highly complex tasks (Müller, 2012). Despite the ongoing trend toward higher education of day-care teachers in Germany, most of them still have completed a vocational training exclusively. That means they have graduated from a school for social pedagogy. This training is more practically oriented than academically informed (Wadepohl, 2019). Daily requirements when working with children in day-care centers demand a high level of competency that includes special knowledge and skills. Acknowledging the importance of practical skills, education for day-care teachers also has to integrate current research and science-based knowledge to promote further professionalization (Wolf, 2015). Trainings for day-care teachers in general should foster competencies that allow them to face present challenges, to solve problems and to implement innovative practice at day-care centers (Fröhlich-Gildhoff et al., 2011). Considering the aforementioned increase in children with refugee experiences, educational practice needs to be not only innovative but also culturally informed. For this reason, the project *Gemeinsam stark durch den Start* (*Stronger together by starting together*) developed a theory-based online training for day-care teachers facing culturally sensitive and informed education as well as an easy-to-apply toolbox to promote intercultural social–emotional learning of all children in day-care centers.

### 1.1. Intercultural sensitivity and intercultural competence

Day-care teachers repeatedly and increasingly act in intercultural overlapping situations with people from different cultural backgrounds and they must be equipped to navigate these challenging situations safely. To do so, they need intercultural competence that enables them to grasp and productively use cultural conditions and that helps them to control influencing factors in their perceptions, judgments, thinking and emotions as well as in their actions (Thomas, 2009; Deardorff and Jones, 2012). Intercultural sensitivity is also relevant in the interaction with children and their families: Without awareness of differences between different cultures, successful intercultural communication and interaction cannot occur (Chen and Starosta, 2000). A high level of intercultural sensitivity is expressed by a deep attitude of ethnorelativism and the ability to think beyond one’s own cultural background. It also includes the ability to consider differences as processes and to adapt adequately in intercultural settings (Chen and Starosta, 2000).

Within the field of intercultural education an ongoing debate addresses the issue if compensatory educational efforts for children with a migration or refugee background promote the acculturation process (Möller, 2012). These compensatory efforts that often focus on the training of language skills and the associated deficit view, are still prominent in educational settings (Åkerblom and Harju, 2019). However, more innovative concepts highlight the problems such as the corroboration of cultural hegemonism and nationalist perspectives inherent in such compensatory and deficit-oriented approaches (Catarci, 2014; Åkerblom and Harju, 2019). Therefore, the present project tried to avoid such elements and did not focus on language skills but instead was based on intercultural reflection and the exchange of intercultural understanding, ideas and practices.

A number of studies have dealt with the promotion of intercultural competence in educational institutions and a recent systematic review has summarized results regarding variables that might influence intercultural competence (Bagwe and Haskollar, 2020). Bagwe and Haskollar (2020) distinguished between intercultural program characteristics and individual or demographic characteristics. Intercultural programs in general should combine self-reflection with training elements and implement different workshop elements like learning reflections, peer support or intercultural interaction for students and professionals. Regarding individual and demographic characteristics experiences of living and working abroad proved to be the most effective way of promoting intercultural competence. Although acknowledging the importance of demographic variables for the development of intercultural competence, the authors conclude that the impact of demographic background must be judged based on the individual case (Bagwe and Haskollar, 2020).

In addition, a recent review investigated efforts to promote intercultural competences in in-service and pre-service teachers (Romijn et al., 2021). The review is based on a general concept of professional development (PD) that integrates the role of individual differences of learners (who), target skills and knowledge of the PD (what), and the strategies used to promote PD, e.g., workshops and implementation of curriculums, into an overall model of PD. The model identifies reflection and corresponding enactment as the basic mechanisms underlying successful PD in teachers. Regarding the promotion of intercultural competence, this review identifies three main elements that might enhance PD in the field of intercultural competence (Romijn et al., 2021): First, the authors highlight the role of context and recommend a team-based strategy with single teachers functioning as counselors in an environment that provides appropriate classroom materials and is supported by a culturally responsible policy. Second, the authors emphasize the importance of targeting teachers´ belief systems and to stimulate reflecting own cultural biases and own ways of interculturally responsive teaching practices. Third, the authors stress the complex relation between beliefs and actions, and conclude based on their findings that sustainable enactment of culturally sensitive teaching practices is still neglected in intervention and evaluation.

### 1.2. Social–emotional learning in intercultural settings

For the professionalization of day-care teachers, intercultural competence and intercultural sensitivity should be linked with knowledge of developmental psychology and developmentally oriented prevention (Scheithauer et al., 2022). Especially children at younger age who are exposed to multiple risks like low social-economical status, poor familiar support and migration background show less social and emotional competencies (Hölling et al., 2008). However, professional support in day-care centers might compensate for some of those risks (Anders, 2013) and might foster children’s social–emotional learning (SEL). The concept of SEL includes five basic areas of skills, namely self-awareness, self-management, social awareness, relationship skills, and responsible decision-making (CASEL, 2013). To promote the positive development of interaction and communication between children in culturally diverse settings, SEL has to be integrated with intercultural knowledge and intercultural contact (Hess et al., 2021). Finally, an effective training to increase professionalization of day-care teachers should include measures to foster intercultural sensitivity in teachers as well as to provide and to train easy-to-apply tools to promote interculturally informed SEL in young children (Romijn et al., 2021).

### 1.3. Teaching intercultural competence for day-care teachers

The first step in developing the intercultural training for day-care teachers was an extensive literature review on intercultural competence in connection with (culturally sensitive) pedagogy as well as a needs analysis based on qualitative interviews with four day-care teachers. A combination of both elements resulted in a theory-driven basic approach, a methodological framework and a didactic-content structure.

The *basic approach* is grounded on the concept of prejudice-conscious education (Preissing and Wagner, 2003) which represents an adapted German version of the “Anti-Bias Approach” (Derman-Sparks and A.B.C. Task Force, 1989). This approach pursues four goals: (1) enable children to develop a self-confident identity, (2) experience diversity and build empathy, (3) stimulate critical thinking about prejudice and discrimination, and (4) work together and actively against discriminatory behavior. This means to also critically question one’s own professional actions and their effects and to commit oneself to justice and to resist injustice (Wagner, 2009). In doing so, day-care teachers also serve as important role models for children (Wagner, 2009). The anti-bias approach was chosen for several reasons. First of all, the anti-bias curriculum has a long history and is well established in early childhood education (ECE). Second, the approach provides a strong foundation in developmental theories, namely the works of Vygotsky (1978, cited from Davidson and Fouts, 2022) who emphasized the role of social interaction in development, and Rogoff (1990, cited from Davidson and Fouts, 2022) who adopted the core assumptions of Vygotsky to apply them to intercultural contexts. Finally, the anti-bias approach and its core components are linked closely to the requirements for a developmentally appropriate practice in early childhood (Sanders and Farago, 2018; Beneke et al., 2019).

**Figure 1:**
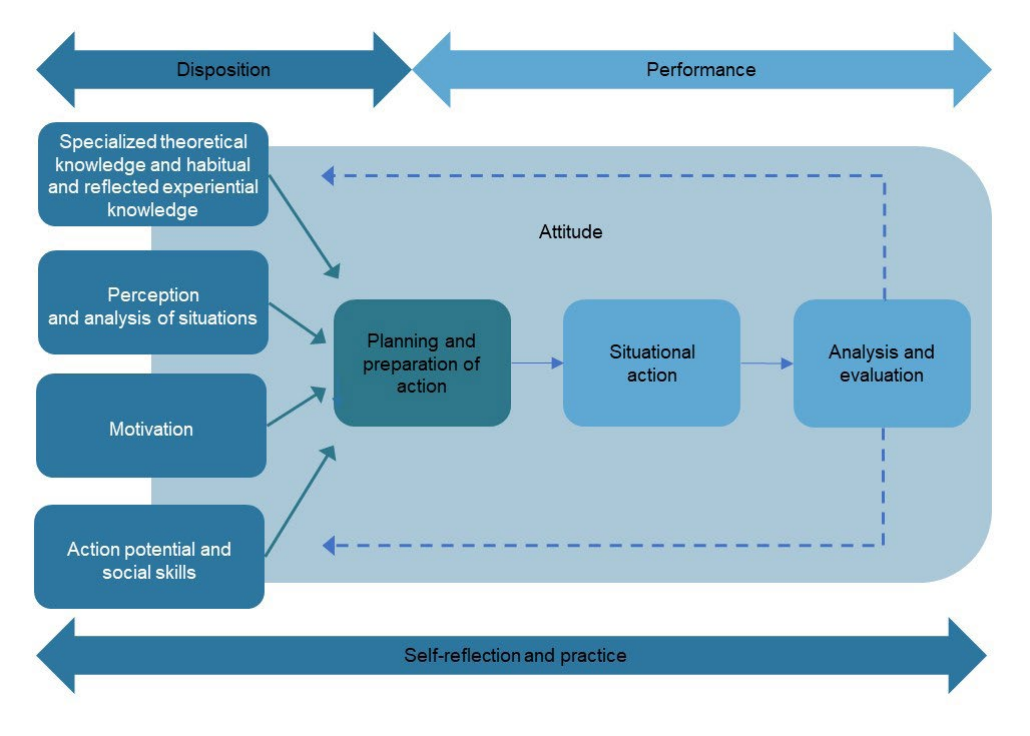
Competence model according to Fröhlich-Gildhoff et al. (2011).

The *methodological framework* is based on a general understanding of competence which is defined as a disposition that enables persons to cope with concrete demands of a certain kind (Klieme et al., 2003). In this case, the specific demand is to cope with the challenges of dealing with refugee children in an intercultural day-care setting. Competence is also expressed in performance, i.e., actual performance in complex situations (ISB - Staatsinstitut für Schulqualität und Bildungsforschung München, 2006). In day-care centers, (intercultural) interaction situations cannot be standardized, they are complex and difficult to predict (Fröhlich-Gildhoff et al., 2011). Training measures should therefore build up competences which, based on (scientific-theoretical) knowledge and reflected experiential knowledge, enable professionals to accept current demands, to solve problems and to shape new and adaptive educational settings in an intercultural context (Fröhlich-Gildhoff et al., 2011; see Figure 1).

The *didactic-content structure* focuses on the working environment of day-care teachers and is structured according to the methodological dimensions of knowledge, attitude, and action. What we know from intercultural trainings in other areas is that a stepwise approach proved to be most successful. In a stepwise approach, basic and more general subgoals must be accomplished before the training addresses more complex or specific subgoals that aim at advanced intercultural competencies. For example, Bennett (1986) reasons that cognitive and emotional perspective-taking comes first (knowledge) so that on this basis empathy can be promoted, which in turn represents the basis for changes in attitude toward cultural diversity (attitude). This can be followed by changes in (planning of) actions in intercultural settings. In the process of developing the present training, contents were derived from the literature review and the needs analysis mentioned above. In the training, each content block is given sufficient time for reflection and practice. The content of the training is summarized in Table 1 along with the dimensions of the methodological framework.

**Table 1:** Structure and objectives of the team training and the in-depths training.

Contents	Methodical dimension according to the competence model
Team training	
Inequality/discrimination in society Knowledge transfer on and sensitization for unequal treatment based on social status, gender, origin and skin color Verbalization of accompanying feelings and reflection in the group Raising awareness of one’s own social identity and reflection on identity attributes that can lead to preferential treatment or discrimination Emotional perspective-taking of disadvantaged persons and their limitations Knowledge transfer on critical whiteness and privileges of insiders	Attitudes (openness, curiosity, tolerance of ambiguity) Knowledge (theorical and reflective knowledge)
2. Inequality/discrimination in childhood Emotional perspective-taking of disadvantaged children Raising awareness of mechanisms of inequality that influence how children think, feel, and act	Attitude (openness, curiosity, tolerance of ambiguity) Reflective knowledge
3. My own power positions Reflection of influence and power on child and parent level Developing an awareness to deal sensitively with one’s own power position and to be able to act as a role model	Attitude (respect, openness, tolerance of ambiguity) Reflective knowledge
4. Our common mission Developing common values and guiding principles that are condensed into a mission (accompanied by the day-care center management) Building common commitment	Attitude (respect, openness, curiosity) Action planning
In-depths Training	
1. Degradation and discrimination in the everyday life of children Reading reports from children who experienced discrimination and racism Verbalization of own accompanying feelings while reading Reflecting on own “take-home” message from the reading-task Emotional perspective-taking of the children and verbalizing their possible feelings and implications Reflection in the group of what the children would have needed from an adult in these situations	Theoretical and reflective knowledge Attitude (openness, curiosity)
2. Dealing with degradation and discrimination Raising awareness of the importance of active action against exclusion Reflection on one’s own role dealing with degradation and discrimination Raising awareness of one’s own possibilities to intervene in cases of discrimination Development of a power-critical and exclusion-critical position and ability to verbalize this position	Knowledge (theoretical and reflective) Attitude (openness, curiosity)
3. Knowledge about early childhood developmental processes Knowledge acquisition of tolerance development and early childhood developmental processed related to prejudice development Knowledge acquisition of preventive strategies against the development of prejudice and for promotion of tolerance development Raising the ability to link the knowledge with the goals and contents of the project	Theoretical and reflective knowledge
4. Consideration of own imprints Reflection of own experiences with stereotypes and imprints for example in children’s books or series from own childhood Reflection on unequal treatment, discrimination, sensitivity in dealing with POC in one’s own institution Planning of concrete implementation steps of what has been learned	Knowledge (theoretical and reflective) Attitude (openness, curiosity, tolerance of ambiguity) Action planning
5. Reflection and knowledge about the building blocks of the toolbox Presentation of the manuals with an example Presentation of single components with exercises Reflection on the (emotional) effects of the implementation on different children and awareness for the children's needs while implementing	Action planning Reflective knowledge Attitude (openness, curiosity)

### 1.4. Research questions

Based on the theoretical background described above, we assume that the training planned in this way will have a positive effect on the three competence areas of knowledge, attitude and action according to Bennett (1986) in day-care teachers and therefore enables them to handle intercultural situations in day-care centers in a professional way. Moreover, we assume that the day-care teachers are then capable of passing these competencies to children, depending on risk-factors in the environment. More specifically, ratings of day-care teachers about environmental risk-factors are thought to be related to ratings of successful intercultural education and professionalization in intercultural competence.

In a mixed methods design, we aim to answer the following research questions:

1. How is the training rated by workshop participants regarding content and benefit for their everyday practice? How does the training advance professionalisation based on ratings from participants? As the training is designed in a stepwise approach as recommended by Bennett (1986), we expect that workshop participants evaluate the training favorably regarding content and benefit for their own work.

2. How does the training and the implementation of training elements into ECE practice have a positive effect on relevant child outcomes? We also expect participants to report positive expectations for outcome on children.

3. How does environmental risk status affect the ratings of participants regarding the effectiveness of the training? We expect that a higher risk status as well as a high level of migration background in the neighborhood predicts greater benefit from the training.

The study will also provide information on mechanisms of action that contributed to the effectiveness of the training from two perspectives: the day-care teachers’ perspective and the trainers’ perspective.

## 2. Materials and methods

The study was designed using a controlled waiting-list-method and was approved by the ethical board of the *DHGS Deutsche Hochschule für Gesundheit und Sport* (German University of Health and Sports). In the intervention group, day-care teachers of 11 German day-care centers with refugee children took part in graded training sessions (team trainings for day-care teachers and in-depth trainings for multipliers). Participants in the control group received a workshop after handing in all questionnaires. In this paper, we only present data based on the training sessions for the intervention group. Firstly, within a pre-post-design all participants were presented with a questionnaire for their training evaluation. Secondly, semi-structured interviews on the training were conducted with multipliers taking part in the in-depth trainings. Thirdly, qualitative interviews were conducted with all trainers (*N* = 4) of the workshops and in-depths trainings to identify which areas emerged as particularly critical or influential in relation to the professionalization of day-care teachers.

### 2.1. Participants

The initial sample recruitment started by contacting all day-care centers of private and public institutions located in the surrounding of large reception centers in the urban area of Hamburg and Augsburg, Germany. Due to the COVID-19 pandemic and the subsequent mandatory switch to a digital format, sample recruitment was expanded to cover the entire country. The project was then advertised online. Interested day-care centers were able to contact the project management independently. Inclusion criterion was the care of refugee or immigrant children between the ages of 3 and 6; exclusion criterion was if this was not the case.

A total of 11 day-care centers participated in the intervention group. Each day-care center sent staff to the team training sessions and up to two day-care teachers each to the in-depth training sessions. Furthermore, all day-care centers managers participated in a special training. However, this training did not focus on professionalization regarding intercultural sensitivity but rather addressed the role of day-care managers within the institutional setting in general. It should not be neglected that managers in general play a major role as providers of opportunities for professionalization within their teams (Fonsén et al., 2023). In this study, leadership training was not the main focus, therefore, this part of the training will not be included in the present analysis.

All an all, day-care centers in the intervention group had a total of around 180 employees. Between four and 17 professionals per day-care center took part in each of the training sessions (*M* = 10.5). Seven professionals participated in qualitative interviews. Demographic data from quantitative questionnaires independent of participation in the team workshops (*N* = 87) show that day-care teacher sample consisted of 76 (87%) female and 10 (12%) male professionals. One staff member indicated gender as “diverse/inter/other.” The professionals age ranged between 18 and 60 years old (*M* = 37, *SD* = 11.63). 70% of all respondents reported own experiences of racism and exclusion. On average, the day-care teachers had been working at their current day-care center for about 7 years (*SD* = 8.0) and had been in charge of their current group of children for about 4 years (*SD* = 4.8). The school qualifications and occupational training of the participating staff is shown in Table 1.

All trainers (*N* = 4) have a background in either psychology or educational science and are experienced in adult education. Each workshop was given by two trainers, with one trainer taking the lead role.

### 2.2. Procedure

In order to examine the effects of the training programs, the evaluation questionnaire was presented within a pre-post-design. The team trainings for the day-care teachers were held between September 6th and October 28th 2021, the in-depth workshops from November 11th to 25th 2021. The day-care teachers were advised to fill out the questionnaire 2 weeks before the training started and as soon as possible after the training ended. If they did not respond within 1 week, they were contacted and reminded to do so in the next days. Data collection was terminated 4 weeks after the last training session. Data was collected through online surveys that were distributed to the day-care teachers via e-mail. The informed consent form and the questionnaires were completed in German by the participants. After all training sessions were finished, semi-structured online-interviews were conducted with in-depth multipliers to gain more profound knowledge about the training. Furthermore, qualitative data about the trainings sessions was derived from semi-structured online-interviews with the trainers of the workshops and in-depths trainings.

### 2.3. Instruments

#### 2.3.1. Evaluation questionnaire

Risk status of day-care center environment was assessed using six items that were answered in a dichotomous fashion. The first question globally asked if there were any problems in the center environment (1 = yes, 2 = no). The remaining five questions dealt with the presence of different risk factors, namely lack of leisure activities, unemployment, drug abuse, high rate of delinquency, high level of environmental neglect. Each item was rated dichotomously (1 = not present, 2 = present). Items were summed up to build a cumulative risk factor, and each item was weighted equally. The risk index could therefore range from 5 (lowest risk) to 10 (highest risk). Due to the main topic of the workshops, we let the teachers rate if there were a lot of families with migration background in the environment of the day-care center.

On a quantitative basis day-care teachers provided direct assessments of the impact the workshop might have on their work after attending the workshop. Day-care center teachers in both conditions (team and in-depths) rated how valuable the workshop was for their day-to-day work. The evaluation included nine aspects of implementation of workshop content:

- Easy to apply elements
- Improvements of work satisfaction
- Improvements of social–emotional competence of children
- No change of child behavior
- Reduction of problematic child behavior
- Problems with non-German speaking children
- Biggest improvement of non-German speaking children
- No improvements of high-risk children
- Biggest improvements for high-risk children

Each of the items was rated on 5-point Likert scale ranging from 1 (“not at all true”) to 5 (“completely true”).

Moreover, the participants of the workshops rated their satisfaction with different elements of the workshop addressing the issue of professionalization. Teachers were asked to report their level of satisfaction with the following statements on a 5-point Likert Scale (from 1 = “very low” to 5 “very high”).

- Level of informational content
- Level of relevance for every-day practice
- Level of benefit for own work

Regarding aspects of professionalization several additional items were included in the workshop evaluation. According to Bennett (1986) these items were categorized based on the dimensions of action, knowledge and attitude (in parentheses). Teachers were asked to report their level of agreement with the following statements on a 5-point Likert Scale (from 1 = “not at all true” to 5 “completely true”).

- I have learned how to implement workshop topics in my day-care center. (action)
- I have refreshed my knowledge about the topic. (knowledge)
- I have extended my knowledge about the topic. (knowledge)
- The workshop taught knowledge about intercultural competence. (knowledge)
- The workshop stimulated self-reflection about my own cultural competence. (attitude)
- The workshop provided new perspectives on the topic. (attitude)

Ratings from teachers were analyzed in a descriptive way. In addition, ratings regarding the workshop evaluation were related to different levels of ratings of risk-status of environments to establish whether the workshops prove their efficacy equally well in different risk settings as reported by the day-care teachers.

#### 2.3.2. Qualitative interviews with multipliers

The semi-structured interview concerning the in-depth training consisted of a series of questions regarding the training itself, the implementation of the toolbox and potential changes (at child level or day-care teacher level). At the end of each interview, participants had the opportunity to add their own comments and feedback. For the purpose of this study, only results regarding statements or changes due to the training are being reported. The in-depth interviews were conducted online and lasted an average of 20:57 min (12:21–28:29 min).

#### 2.3.3. Qualitative interviews with trainers

The semi-structured interview for the trainers focused on the trainers’ impression of the workshops and in-depths trainings. They were asked which elements of the training they thought worked well, whether there were differences between the training groups and whether there were difficulties and how they dealt with them, if any. The trainers’ interviews were also conducted online and lasted on average 62:09 min (50:08–85:50 min).

### 2.4. Data analysis

Quantitative data was analyzed using SPSS version 28 (IBM, 2022). Descriptive data are reported. This was done to describe the overall satisfaction with the program addressing research questions one and two. In order to address research question three and to compute the relation between migration background and cumulative risk factors and different ratings Pearson correlations were used. In order to control for violations of assumptions related to the use of Pearson correlations a bootstrapping procedure was applied. Data were estimated based on 1,000 bootstrapping samples.

In addition to bivariate analysis several multiple regressions were conducted. This was done to learn more about the concurrent predictive value of different risk factors in the environment for workshop evaluation addressing research question three. Therefore, in a first set of regression analyses we used the single risk factors and not the cumulative risk as predictors and the different parameters of workshop evaluations as outcomes. In a second set we used the cumulative risk index and the migration background as predictors and workshop evaluation as outcomes. To reduce the number of outcome variables composite indicators were computed as means of the single indicators of each workshop evaluation topic if possible (implementation of training elements, improvement of professionalization, satisfaction with training elements). Improvements of professionalization were summarized according to areas suggested by Bennett (1986, see Methods section) To account for possible heteroscedasticity due to the nested data structure and considering the rather small sample size, robust standard error estimators were used.

For the analysis of qualitative data, all interviews were recorded and transcribed. The procedure for multipliers’ interviews and trainers’ interviews were the same, all interviews were worked through using qualitative content analysis and categorized using a derived code book.

## 3. Results

### 3.1. Quantitative analysis

Altogether, 105 persons took part in the pre-trainings or the post-training questionnaires. A total of 76 participants filled in the pre-training evaluation questionnaires, 65 participants provided ratings for the post-training questionnaires with 40 participants participating in both questionnaires. Regarding the additional in-depth-trainings, 16 from a total of 19 participants provided pre-and post-training data. The weak overlap between pre and post data resulted from the fact that the workshops were distributed over several days. Due to pandemic related issues, e.g., work overload and sick leave, participation in trainings sessions as well as response rates regarding evaluation questionnaires varied. Therefore, the overlap between pre-and post-data was rather low.

In a first step, descriptive parameters regarding the subjective individual ratings of at-risk status of the day-care center location were computed as a sum score for participants. These questions were asked in the questionnaire before the training took place. From these 76 participants, 33 (43%) expressed no special problems in their day-care center environment. The mean cumulative risk index (based on six items) was 7.54 (with a possible range from 6 to 12), with 51% of the teachers reporting no risk factor at all and 16% of the teachers reporting more than two risk factors within the environment of their day-care center.

The descriptive results from the post-training assessment are reported in Table 2.

**Table 2:** Descriptive values of ratings regarding improvements in professionalization of day-care teachers attending the team-training and in-depth training.

Item	Type of training			
	Team (*n* = 65)		In-depth (*n* = 16)	
	*M*	*SD*	*M*	*SD*
Implementation of training elements				
Easy to apply elements	4.09	0.88	–	–
Improvements of work satisfaction	3.86	1.01	3.81	0.91
Improvements of child social-emotional competence	4.17	0.86	4.19	0.83
No change of child behavior	2.34	1.05	2.31	1.01
Reduction of problematic child behavior	2.81	1.21	3.00	1.10
Problems with non-German speaking children	2.85	1.02	2.87	1.15
Biggest improvement of non-German speaking children	3.65	0.94	3.50	0.89
No improvements of high-risk children	2.19	1.02	2.19	1.11
Biggest improvements for high-risk children	3.25	0.94	3.44	0.89
Improvement of professionalization				
Learned how to implement workshop topics in my day-care center (action)	3.78	0.99	4.00	0.97
I have refreshed my knowledge about the topic (knowledge)	4.00	1.02	4.00	0.85
I have extended my knowledge about the topic (knowledge)	3.91	1.20	4.13	0.74
Workshop taught knowledge about intercultural competence/ values regarding intercultural sensitivity (knowledge)	4.03	1.00	4.33	0.82
Workshop stimulated self-reflection about own cultural competence (attitude)	4.18	1.01	4.53	0.52
The workshop provided new perspectives on the topic (attitude)	3.83	1.21	4.27	0.80
Satisfaction with training elements				
Level of informational content	3.88	0.92	3.94	1.06
Level of relevance for every-day practice	3.84	1.05	3.38	1.41
Level of benefit for own work	3.94	1.03	3.87	1.20

Ratings range from 1 to 5.

The correlations between cumulative risk indexes, indicator of level of migration background within the day-care center environment and ratings of the workshop revealed the following results (see Table 3; in the table only significant correlations are shown).

**Table 3:** Correlations between parameters of day-care center environment and ratings of the trainings’ content and implementation (Pearson’s r with bootstrapping).

Item	Migration background (no/yes)		Cumulative risk factor	
	*r*	95% CI	*r*	95% CI
Implementation of workshop elements				
Easy to apply elements	−0.28	[−0.55 −0.02]	−0.21	[−0.53 0.17]
Improvements of work satisfaction	−0.43**	[−0.61 −0.22]	−0.38*	[−0.70 −0.02]
Improvements of child social-emotional competence	−0.34*	[−0.56 −0.12]	−0.32*	[−0.60 0.00]
No change of child behavior	−0.10	[−0.42 0.31]	−0.25	[−0.10 0.57]
Reduction of problematic child behavior	−0.07	[−0.41 0.29]	−0.30	[−0.02 0.57]
Problems with non-German speaking children	−0.01	[−0.33 0.36]	−0.29	[0.03 0.52]
Biggest improvement of non-German speaking children	−0.19	[−0.52 0.16]	−0.05	[−0.31 0.33]
No improvements of high-risk children	−0.31	[−0.01 0.65]	−0.45**	[0.18 0.70]
Biggest improvements for high-risk children	−0.15	[−0.48 0.29]	−0.24	[−0.04 0.50]
Improvement of professionalization				
Learned how to implement workshop topics in my day-care center (action)	−0.19	[−0.47 0.14]	−0.00	[−0.35 0.36]
I have refreshed my knowledge about the topic (knowledge)	−0.14	[−0.36 0.13]	−0.07	[−0.47 0.27]
I have extended my knowledge about the topic (knowledge)	−0.39*	[−0.58 −0.17]	−0.23	[−0.58 0.09]
Workshop taught knowledge about intercultural competence/values regarding intercultural sensitivity (knowledge)	−0.27	[−0.51 0.00]	−0.08	[−0.45 0.23]
Workshop stimulated self-reflection about own cultural competence (attitude)	−0.49**	[−0.69 −0.30]	−0.36*	[−0.67 −0.05]
The workshop provided new perspectives on the topic (attitude)	−0.60**	[−0.74 −0.45]	−0.30	[−0.63 0.04]
Satisfaction with workshop elements				
Level of informational content	−0.23	[−0.46 0.05]	−0.11	[−0.48 0.18]
Level of relevance for every-day practice	−0.46**	[−0.65 −0.23]	−0.32*	[−0.64 0.02]
Level of benefit for own work	−0.53**	[−0.69 −0.34]	−0.21	[−0.59 0.10]

*p < 0.05; **p < 0.01. The sample for this analysis consisted of 40 teachers who provided valid data before and after the workshop. Migration background was coded 1 = no and 2 = yes. A Bootstrapping procedure with 1.000 samples was applied in the analysis.

Results reveal that high levels of migration background in the day-care center environment is related to lower levels of expected success in implementing the workshop content. The same pattern evolved regarding self-rating of improvements of professionalization and satisfaction with the trainings’ content.

Likewise, higher ratings of risk status of the day-care center environment provided by day-care teachers were related to lower ratings of the chance of improvement for children as well as lower ratings of improvement regarding own professionalization. In addition, higher risk status was associated with lower satisfaction regarding the overall training contents and a lower expectation that the training will be effective for high-risk children.

Additional regression analyses with robust estimation of standard errors revealed that no single risk factor predicted ratings of implementation of training elements. However, using cumulative risk and migration background as predictors results show that lower ratings of migration background predicted higher ratings of implementation success *b* = −0.32, *t*(37) = −2.07, *p* = 0.045. Almost the same pattern emerged regarding ratings of attitude change as one indicator of professionalization. Here in both regressions (single risk indicators vs. cumulative risk as predictors) higher ratings of a migration background environment predicted lower self-ratings of attitude change [*b* = −0.99, *t*(35) = −3.13, *p* = 0.002 and *b* = −1.02, *t*(37) = −4.03, *p* < 0.001]. Ratings of knowledge improvements were not predicted by any risk indicator. Lower ratings of improvements in action strategies based on workshop participation were predicted by higher rating of a migration environment when cumulative risk was used as a second predictor [*b* = −0.79, *t*(37) = −2.25, *p* = 0.030]. Overall satisfaction with workshop elements was equally and in the same direction as previous outcomes only predicted by the level of migration background in the environment [*b* = −0.71, *t*(35) = −2.23, *p* = 0.032 and *b* = −0.70, *t*(37) = −2.59, *p* = 0.013].

### 3.2. Qualitative analysis: interviews with in-depth multipliers

Analysis of the interviews with in-depth multipliers (M1-M7) resulted in a total of 12 categories. Most of the categories revolve around the material and the toolbox as well as the implementation of the toolbox in day-care centers and problems with the implementation due to COVID (see Table 4). One category focuses on elements of the training sessions and workshops. These results are reported in detail.

**Table 4:** Categories and explanations of categories identified in interviews with in-depths multipliers.

Category	Explanation
Structure of the training	Feedback on the structure of the training and on the expectations of the training
Accompaniment/Support	Feedback on the accompaniment offered in the project, especially on case of questions during implementation
Evaluation of the modules	Feedback from day-care teachers on the modules themselves
Group organization	Implementation problems that have arisen dure to the organization of the groups in the day-care centers, for example, due to COVID-related emergency care or due to implementation with larger groups
Reception by the children	Reports on the children’s feedback on the individual components of the project and children’s perceived understanding by the day-care teachers
Structuring of the materials	Feedback on the design and structuring of the materials themselves, as well as in relation to the instructions and flexibility of the modules
Implementation	Experiences and adaptations in the actual implementation
Understanding of the toolbox	Feedback on the understanding of the toolbox
Suggestions	Proposals for future revisions and adjustments
Time allocation	Feedback on the implementation of the project within the time resources of the day-care center
Timing of the modules	Feedback on the implementation of the modules in the time allotted for the respective module
Other	One interviewee explicitly emphasized the importance of the project itself

In-depth multipliers noted that in their opinion the training covered a lot of theoretical input and at the same time did not cover certain elements sufficiently (M4 “So sometimes I had the feeling that it was just a bit theoretical”; M5 “Instead of the 2nd unit in the in-depth training, it would be better to work on the modules more so that you can find your way in better”). In particular, in-depth multipliers criticized that it did not include enough knowledge about the toolbox, how to apply it correctly and how to deal with specific situations (M6 “For us, however, it also somehow had a lot to do with what was not done in the in-depth training”). They reported that they had to work out a lot for themselves after the training sessions (M6 “We had to spend a lot of time working on it ourselves afterwards”) and therefore they felt uncertain regarding different aspects of the toolbox and its implementation (M6 “I was so unsure because I understood almost […] because we have never done anything like that here either”). Several times the wish was expressed to get to know the toolbox better during the training.

In-depth multipliers also reported changes in their teams. In their opinion a lot of self-reflection had taken place and this had triggered change processes in the whole team. The training sessions strengthened them as a team due to the exchange of very private opinions and emotions they revealed to one another during the trainings and thus made their “system” stronger and changed the cohesion in the team. In addition, they noticed that they had been sensitized to discrimination, prejudices and stereotypes. They reported that before the training, there were many things they did not think about, for example whether a certain situation excluded one or some of the children, but after the training, they began to pay more attention in these critical situations.

### 3.3. Qualitative analysis: interviews with trainers

Analysis of the interviews with trainers (T1-T4) showed three major categories: (1) Evaluation of the trainings in terms of content and structure, (2) Online implementation and (3) Aspects of professionalization of day-care teachers (see Table 5).

**Table 5:** Categories and explanations of categories identified in interviews with trainers.

Category	Explanation	Number of mentions
Evaluation of the trainings in terms of content and structure	Positive and negative aspects regarding the training content and structure, such as material and exercises for training sessions, duration of training sessions and division of training units	21
Online implementation	Technical problems with the training platform, limited possibilities for exchange online in between the training units, challenges due to the fact that some participants shared a computer	12
Aspects of professionalization of day-care teachers	Achieving new perspectives, especially with exercises that emotionally evoked participants, trust in the team necessary for opening up, challenge of questioning one’s own, possibly racist, thought patterns	20

Regarding (1), trainers rated the trainings as a good fit regarding the structure and content (T3 “I thought the topics were well chosen and I also thought the division was actually good, just as it was”). They deemed the duration of the training sessions a good fit as well as the duration of training units and the division of these units. At the same time, some of the training units might be improved by shortening their duration (T1 “I would shorten it a bit in terms of content; that would really loosen things up”). All trainers stated that it is of great importance to encourage participants to engage in the exercises and to emotionally connect with the topic. They also noticed that participants wanted more concrete action instructions for the toolbox and for critical situations as well as a more concrete practice of the individual components (T2: “In the training, it became clear that they would have needed much more specific information about the components, that is, what they are supposed to work on with the children. Many questions remained open”).

For (2), trainers focused on problems due to the platform, e.g., troubles with log in processes, and on problems arising from the fact that in some training sessions several participants shared one computer (T3 “Technically, we often had many problems, in data transmission, so it was often choppy, people got kicked out, were hard to hear or the image did not work”). For that reason, there were side conversations in some of the sessions that not all participants could partake in (T1 “With the groups that sat in front of one laptop together, it never worked that well”). Furthermore, trainers missed the possibilities for exchange among the participants in between the training units that on-site training sessions offer.

Concerning (3), trainers noticed that especially those sessions that emotionally evoked participants led to taking new perspectives (T2 “What consistently worked well for everyone was that we continually focused strongly on emotions, that they trace and write down their emotions. This was super important because it could create sympathy from it. That was also important for their learning processes”). For this to happen, teams needed to be able to trust one another (T4 “I believe that the team must be very familiar with each other, so that they can also deal openly with each other”). From the trainers’ point of view, the respective team lead held the key position: If the team lead was positive and open toward the training and supported its implementation in the day-care center, it was easier for the teams to implement the exercises in depth and to develop further (T3 “The team lead can obviously somehow also set an example”). This was especially true for the challenge arising due to questioning one’s own, possibly racist, thoughts patterns. Furthermore, this seems to have been particularly difficult for those participants who described themselves as interculturally aware and competent (T1 “There was really such a big oppositional stance”). Some trainers also noted that the process was easier for participants who already had prior knowledge of the subject.

## 4. Discussion

The present research aims at evaluating the impact of an intercultural training designed using the competence model for ECE (Fröhlich-Gildhoff et al., 2011). Therefore, effects on the three areas of knowledge, attitude and action in day-care teachers are discussed from the day-care teachers’ perspective and the trainers’ perspective.

Quantitative data based on retrospective standardized self-report questionnaires reveal that overall assessment of the training was positive, especially for improvement of professionalization and implementation of training elements. However, there are mixed results on satisfaction with training elements: Regarding professionalization of action, day-care teachers reported improvements in their ability to implement the trainings’ topics into day-care center practice. Furthermore, they stated that refreshing and extending their knowledge about intercultural competence and sensitivity were important parts in the training. Looking at attitudes, the training also led to intense self-reflection about their own intercultural competences and provided new perspectives to day-care teachers. So, in general, the intercultural training addressed all three areas of competence (Fröhlich-Gildhoff et al., 2011) based on ratings from the involved day-care teachers. Concerning their satisfaction with training elements, day-care teachers valued the trainings’ level of informational content and relevance for their every-day practice as well as benefits for their own work. According to the evaluation, training elements seem to have been easy to apply and brought improvements of work satisfaction. The day-care teachers highlighted the training elements’ potential to improve the children’s social–emotional competence, but expressed reservations about the workshops impact on children’s problematic behavior. Greenberg and Abenavoli (2016) point out that certain treatment effects may show immediately after a universal intervention (e.g., the reduction of aggression and improvement of social competence after a SEL intervention), but prevention effects often only occur after some time. Similar findings result from the evaluation of the PATHS curriculum, a program for social–emotional learning in schools (Kusché and Greenberg, 2012), as some changes did not show at post-test, but instead unfolded at one- or two-year follow-ups (Riggs et al., 2006; Malti et al., 2011; Crean and Johnson, 2013). Therefore, long-term follow-ups seem necessary to detect effects like these (Greenberg and Abenavoli, 2016). Also, in the education field even small effects could be considered as large according to Lipsey et al. (2012). All in all, ratings of day-care teachers confirm the universal approach of the workshops to be useful for all children, either in low or high risk situations regardless of their native language.

Next, we analyzed how different environmental risk factors are related to workshop assessments. It is a well-known phenomenon that risk groups with a high need for prevention have low participation rates in preventive programs (Ehlen et al., 2022). This also seems true for institutions in high-risk settings: Overall, only 16% of participating day-care centers reported more than two environmental risk factors. Bivariate analyses and results from multiple regressions show that day-care centers with a high migration status in their environment had lower expectations for the training to improve work satisfaction and children’s social–emotional competences. They also did not feel that the workshops extended their knowledge about interculturality, stimulated their self-reflection or provided new perspectives. In addition, levels of relevance for every-day practice and benefits for work were rated low. An examination of environmental risk factors reveals a number of similarities, but also some differences. In summary, day-care teachers who subjectively reported living in a high-risk environment rated the workshop to be less effective regarding their extension of knowledge, stimulation of self-reflection or getting new perspectives. They also deemed the training to be of lower relevance for their every-day practice and benefits for their own work. Higher numbers of perceived risk factors in the environment were related to lower expectancies of training effectiveness for children. High migration environment was stronger related to training assessment than other risk factors in the environment. All in all, that indicates that trainings should be extended to include tailored elements for high-risk environments. It should be noted that a high-risk intervention strategy can only be effective when prior screening for risk status is accurate (Greenberg and Abenavoli, 2016). Even though, a considerable uncertainty of fit remains, which justifies a more universal and broader approach (Merry and Spence, 2007; Shamblen and Derzon, 2009). Therefore, to foster professionalization in pedagogical staff, more effort should be invested to convince those in high-risk settings of the effectiveness of universal intervention programs. This might lead to higher motivation to implement program elements und also improve work-based self-efficacy. In addition, the results regarding the impact of risk factors and level of migration in the environment on ratings of program effectiveness might still reflect a deficit view of day-care teachers that was not addressed strong enough within the workshop elements. Although the present project tried to avoid any implicit or explicit promotion of a deficit view of migration status, for day-care teachers the need for compensatory education facing risky environments seemed to remain a prominent cognitive scheme that should be considered in more detail in future adaptations of the training concept.

Similar to quantitative data, qualitative data obtained from in-depths multipliers show a positive evaluation of the program with the necessity to readjust certain training elements and also allow a deeper insight into their impressions: They would have liked to gain more (theoretical) knowledge about all elements of the toolbox. In their opinion, this would lead to a straightforward start into implementation without uncertainties and to being able to deal with specific situations. Despite many difficulties, especially with technical and organizational issues, the training elicited appropriate self-reflection, brought the respective teams closer together and raised (more) awareness of discrimination, prejudice and stereotypes.

In line with results from the recently conducted review by Romijn et al. (2021) in the present study, the transfer from workshop contents regarding intercultural knowledge and beliefs to enactment in every day ECE practice was judged as difficult by participating teachers, although they were provided with teaching materials and guided in implementing these materials in their institutions. Again, limited resources due to pandemic conditions resulting in high work-load and constant pressure to adapt to new working conditions might have hindered a more successful enactment of interculturally responsive practices.

Problems arising from technical and organizational difficulties were also mentioned by trainers in their interviews, as well as the impossibility for close interaction between workshop participants and between trainers and participants. They also pointed out the need for certain improvements, especially because they noted that participants had wanted more concrete action instructions and practice for the toolbox as well as for critical situations at work. This is in line with findings from several studies about workshops and practical material on intercultural and interreligious education in day-care centers: Practice-oriented and interactive training courses were rated as more popular (Wolking and Vestweber, 2020).

Overall, the trainers rated the trainings as well-structured and well-suited in terms of content. For them, emotional perspective-taking is the core component for an effective training with trust between participants being the most important prerequisite. Whether trust-building within the teams worked was perceived to be dependent on the team lead. The interviews also revealed some explanations for the day-care teachers’ low estimation of effectiveness in high-risk day-care centers: The trainers found that participants who already rated themselves as interculturally aware and competent struggled most when questioning their own believes and admitting that they may need to scrutinize their own stereotypes and attitudes. Similar results were reported in several studies on intercultural and interreligious education in day-care centers (workshops and practical work): In pre-post comparison, subjective knowledge remained unchanged, intercultural perspective-taking improved slightly and intercultural awareness as well as openness decreased significantly, indicating a weak effect (Gräbs Santiago and Vestweber, 2020). According to Gräbs Santiago and Vestweber (2020), the latter can be explained by the fact that the intensive examination of intercultural topics can lead to a defensive attitude. If a workshop was multi-day, held in presence and provided space for informal exchange, positive results for intercultural perspective-taking were more pronounced (Gräbs Santiago and Vestweber, 2020). Therefore, trainers should be prepared to be confronted with these defensive attitudes and be able to intervene and trainings should allow the time for letting these processes happen.

It should also be discussed whether day-care teachers in high-risk environments are so used to large scale problems that they might overlook small improvements. Still, the program might have a substantial overall benefit. In universal prevention, small improvements in high-risk populations have the potential to impact positively on population level and do not only affect the participating individuals, but also indirectly a larger spectrum, therefore non-participating individuals may profit from the preventive program (Greenberg and Abenavoli, 2016). This might be true for the intercultural context as well. It is therefore important to draw attention to small changes when working with day-care teachers to improve their professionalization. As recommended by Romijn et al. (2021) the present program addresses issues like self-reflection and provided helpful teaching material for ECE professionals that might help to implement sustainable strategies for intercultural education. However, as the program structure regarding workshop content was rather standardized at this stage of development, qualitative and quantitative results show that the assessment of the environmental context should be integrated into workshop planning to provide more tailored concepts for single institutions. Discussing the composition of the workshop elements to foster professionalization, a stronger focus on a critical self-reflection of the compensatory and deficit view regarding children with a migration and refugee background would probably have helped to improve workshop effectiveness (Sales et al., 2011).

Finally, it should be highlighted that to our knowledge among all scientifically sound and evidence-based programs for ECE as listed by CASEL^1^ and the German database for prevention programs,^2^ no program focuses on the combination of SEL with intercultural topics. Also, the main focus of existing programs remains on the development of children and not on empowering day-care teachers to work in intercultural overlapping situations based on a culturally informed practice.

### 4.1. Limitations

There are several limitations of this pilot study that need to be considered: First, serious implementation problems were caused due to the COVID-19 pandemic. All participating day-care centers were affected by pandemic-related closures and/or emergency and reduced care as well as with staff shortages due to illness during the project period. As a consequence, a rather high drop-out rate of participants and a variation of day-care teachers attending training sessions, participating in surveys and finally implementing the toolbox in their institutions resulted. We assume that this inconsistency disrupted team processes as Wolking and Vestweber (2020) point out. Participation in an entire training series in a fixed group enables collegial cohesion, increases familiarity and stimulates a deeper reflection process. Furthermore, training measures are more effective when the majority of the day-care teachers participated as a team and the training was experienced as a collaborative project (Boschki and Schweitzer, 2020). These serious implementation problems may have negatively influenced the prevention and intervention success (Durlak et al., 2011) and also the professionalization of day-care teachers.

Second, inconsistencies in participation due to the COVID-19 pandemic also affected quantitative data collection, e.g., when due to illness or work overload day-care teachers missed to fill in all questionnaires. Despite prolonged times to fill in the questionnaires and multiple reminders from the project staff members, response rate of day-care teachers regarding quantitative data remained rather low. In addition to low response rates, a high variation in participation in surveys negatively affected analysis of quantitative data as the number complete datasets was rather low compared to the overall number of participants.

Third, the online context caused some interferences with the proper delivery of the online training: In several cases, day-care teachers had to share a device. This led to the risk of distraction and inattention within the group and may have consequently limited the readiness for intensive and emotional reflection processes.

Fourth, results were limited to self-reports from day-care teachers and interviews with in-depths multipliers and workshop trainers. A multi-informant approach including children and parents was not applicable due to restrictions regarding the project conditions: Apart from difficulties to receive valid information from young children, the project framework was restricted to the day-care center staff and it was not possible to include parental reports. In addition, due to the COVID-19 pandemic and the fact that all project parts were delivered online, it was not possible to visit day-care centers to collect observational data. However, addressing improvements of professionalization, day-care teachers self-assessment and professional assessment of trainers represent a valid source of information.

Fifth, the inclusion of a convenience sample of day-care institutions led to the problem that participating day-care centers reported rather low levels of risk in their environment. This is in line with the prevention paradox as mentioned above (Ehlen et al., 2022). In addition, most day-care teachers also reported rather high levels of individual job satisfaction. Therefore, future studies should include a more heterogeneous sample regarding environmental risk factors such as high rates of unemployment or low levels of job satisfaction within the day-care center staff.

### 4.2. Conclusion

Applying the general competence model explained above (Fröhlich-Gildhoff et al., 2011) to the results, it becomes apparent that developments have taken place in all three core areas: (theoretical and reflexive) knowledge, attitude, and actions. In accordance with current research, this study revealed the importance of combining scientific and practical knowledge (Dewe and Otto, 2015). The main focus of trainings should be on emotional self-reflection (Müller, 2012) and detailed hands-on exercises for specific situations in order to manage them in an innovative way (Fröhlich-Gildhoff et al., 2011)—in this case intercultural situations that need to be solved in a culturally informed manner. Besides improving topic relevant knowledge and skills, day-care teachers, especially in high-risk environments, should be encouraged to pay more attention to small improvements and to acknowledge the benefits resulting from those changes in the long run. While it is possible that deepening or intensifying the training may have additional benefits for the effectiveness of the training for day-care centers in high-risk environments, one has to keep in mind that a universal approach has the potential to reach and benefit a wide target population. Therefore, this study provides first insights into the value of this training program for the professionalization of day-care teachers in intercultural working and education situations.

## Data availability statement

The original contributions presented in the study are included in the article/supplementary material, further inquiries can be directed to the corresponding author.

## Ethics statement

The studies involving human participants were reviewed and approved by the ethical board of the DHGS Deutsche Hochschule für Gesundheit und Sport (German University of Health and Sports). The patients/participants provided their written informed consent to participate in this study.

## Author contributions

UB, CE, and MH contributed to the conception and design of the study. CE collected the data. MH performed the quantitative analyses and reported results. UB analyzed the qualitative data and reported results. UB and CE wrote the first draft of the manuscript except the methods section. MH and UB wrote the first draft of the methods section. All authors contributed to the final and submitted version of the manuscript, read, and approved the submitted version.

## Funding

This project was funded by the German Federal Office for Migration and Refugees with a grant from the Asylum, Migration and Integration Fund of the European Union via Plan International Germany.

## Conflict of interest

The authors declare that the research was conducted in the absence of any commercial or financial relationships that could be construed as a potential conflict of interest.

## Publisher’s note

All claims expressed in this article are solely those of the authors and do not necessarily represent those of their affiliated organizations, or those of the publisher, the editors and the reviewers. Any product that may be evaluated in this article, or claim that may be made by its manufacturer, is not guaranteed or endorsed by the publisher.

## Footnotes

^1^ https://pg.casel.org/review-programs

^2^ https://www.gruene-liste-praevention.de/
